# Electromagnetic exposure analysis of the subway passenger under the civil communication system radiation

**DOI:** 10.1371/journal.pone.0300049

**Published:** 2024-03-11

**Authors:** Wen-Ying Zhou, Xi-Yu Zhang, Mai Lu

**Affiliations:** Key Laboratory of Opto-Electronic Technology and Intelligent Control of Ministry of Education, Lanzhou Jiaotong University, Lanzhou, China; Chitkara University, INDIA

## Abstract

In order to assess the electromagnetic exposure safety of passengers under the civil communication system of the subway, the radio-frequency (RF) electromagnetic environment of subway carriage is established by using COMSOL Multiphysics software, it includes a 1-1/4 " leaky coaxial cable (LCX1) and a 1-5/8" leaky coaxial cable (LCX2), which are designed to be the exposure sources, and twelve passengers at different position. The electromagnetic environment model has been verified through field measurement. The exposure dose distribution of twelve passengers is compared and analyzed, when LCX1 and LCX2 works respectively. The simulated results show that, to compare with LCX2, the electromagnetic dose absorbed by the passengers is reduced by 9.19% and 22.50% at 2100 MHz and 2600 MHz respectively. The specific absorption rate (SAR) of passengers obtains the maximum value of 1.91×10^−4^ W/Kg and the temperature rise to 0.214 K when the LCX1 works at 3400 MHz. By comparing with the public exposure limitation of the International Commission of Non-Ionizing Radiation Protection (ICNIRP), it demonstrates the electromagnetic exposure safety of the passengers under the civil communication system. More importantly, the proposed LCX1 not only could add the 5G signal cover but also lower the SAR absorbed by the passengers, which indicates that the public electromagnetic exposure dose could be reduced by adjusting the radiation performances of exposure source, which provide a new way for electromagnetic protecting.

## 1.Introduction

In recent years, as a green and environmental-friendly transportation, the subway has become the main trip model of urban public. The communication system in the subway includes dedicated communication system and civil communication system. The leaky coaxial cable (LCX), as the radiation terminal for civil communication systems, is used to meet the mobile communication needs of passengers. There are two main methods for offering 5G coverage in subway scenarios, one is adding a new LCX with 5G bands to work together with the existed LCX with 4G bands. Another method is to directly build a LCX with full communication bands for the civil communication system. Thus, the increasing communication requirements make the electromagnetic environment in closed subway tunnels become more complex, which highlighted the signification of the electromagnetic exposure safety assessment.

The potential health risk which caused by RF exposure draw the public attention. Many organizations such as Institute of Electrical and Electronics Engineers (IEEE) and the ICNIRP have established guidelines and standards based on peak specific absorption rates, to limit the exposure of the electromagnetic field [1–3]. Radio-frequency electromagnetic field (RF-EMF) radiation may cause health damage to the human body [4–8]. When exposed to RF-EMF, the energy absorbed by biological tissues is converted into heat which leads to an increase in temperature of the tissues. A significant increase in temperature can lead to adverse reactions [9] in the human body.

At present, there have been many studies on human RF electromagnetic exposure caused by different antennas [10–15]. In reference [16], an electronic band gap (EBG) applied to wearable antennas was designed to reduce the specific absorption rate. Reference [17] analyzed the SAR and temperature increase of the human head caused by mobile phone radiation under different usage modes. Reference [18] conducted a numerical analysis on SAR and temperature distribution of a child’s head model under 900MHz mobile phone radiation. In addition to antenna radiation research, there are also researches on the electromagnetic environment of rail transit [19–21]. Reference [22] evaluated the electromagnetic exposure safety of passengers in fully loaded high-speed train carriages. Reference [23] conducted numerical simulation on the electromagnetic dose of passengers in subway platforms. However, the studies for radio frequency electromagnetic environment of closed subway tunnels and corresponding electromagnetic protection methods are rare, which is the research purpose of this paper.

Since the exposure dose absorbed by the passengers can’t be measured directly, our study assesses the RF electromagnetic exposure of the passengers by using numerical calculation. SAR is a dosimetric quantity defined as the electromagnetic radiation energy absorbed by a unit mass of material per unit time, which has been widely used to evaluate the electromagnetic energy absorbed by a unit mass of human tissues [24–26].


$$SAR=\frac{\sigma }{2\rho }{\left|E\right|}^{2}$$ (1)

In the formula, ***E*** is the electric field intensity (V/m), and σ is the conductivity (S/m), *ρ* is the tissue density (kg/m^3^).

For solving the thermal problem, the temperature rise of the head models is obtained by solving Pennes’ bio-heat equation. The transient bio-heat equation [27] can be written as:

$$\rho C\frac{\partial T}{\partial t}=\nabla ∙(k\nabla T)+\rho_{b}C_{b}\omega_{b}(T_{b}-T)+Q_{met}+Q_{ext}$$ (2)

Where *ρ* represents the tissue density (kg/m^3^), *C* represents tissue specific heat capacity (J/(kg·°C)), *k* represents thermal conductivity(W/(m·°C)), *T* represents tissue temperature (°C), *T*_*b*_ represents blood temperature (°C), *ρ*_*b*_ represents blood density (kg/m^3^), C_b_ represents blood specific heat capacity (J/(kg·°C)), *ω*_*b*_ represents blood perfusion (1/s), Q_met_ represents metabolic heat source (W/m^3^), Q_ext_ represents external heat source (W/m^3^).

The main content of our work includes that, 1) As the exposure source of civil system, LCX1 is designed and optimized, which can cover the 700 MHz~3600 MHz frequency band to satisfy 3G ~ 5G communication requirements. Through comparing with the performance of LCX2 (800 MHz~2700 MHz), the proposed LCX1 have lower transmission loss and higher couple loss. 2) The electromagnetic exposure safety of passengers is assessed. The RF-EMF environment in the subway carriage is simulated by using COMSOL Multiphysics software, and the electromagnetic dose absorbed by passengers at different positions and input powers are analyzed by calculating SAR and temperature rise. 3) A new approach for electromagnetic protection is provided. Comparing the impact on passengers radiated by two LCXs, it further indicates that the public electrical exposure dose absorbed by passengers could be reduced by adjusting the radiation source.

## 2.Numerical calculation model

### 2.1 Exposure source model

**Fig 1:**
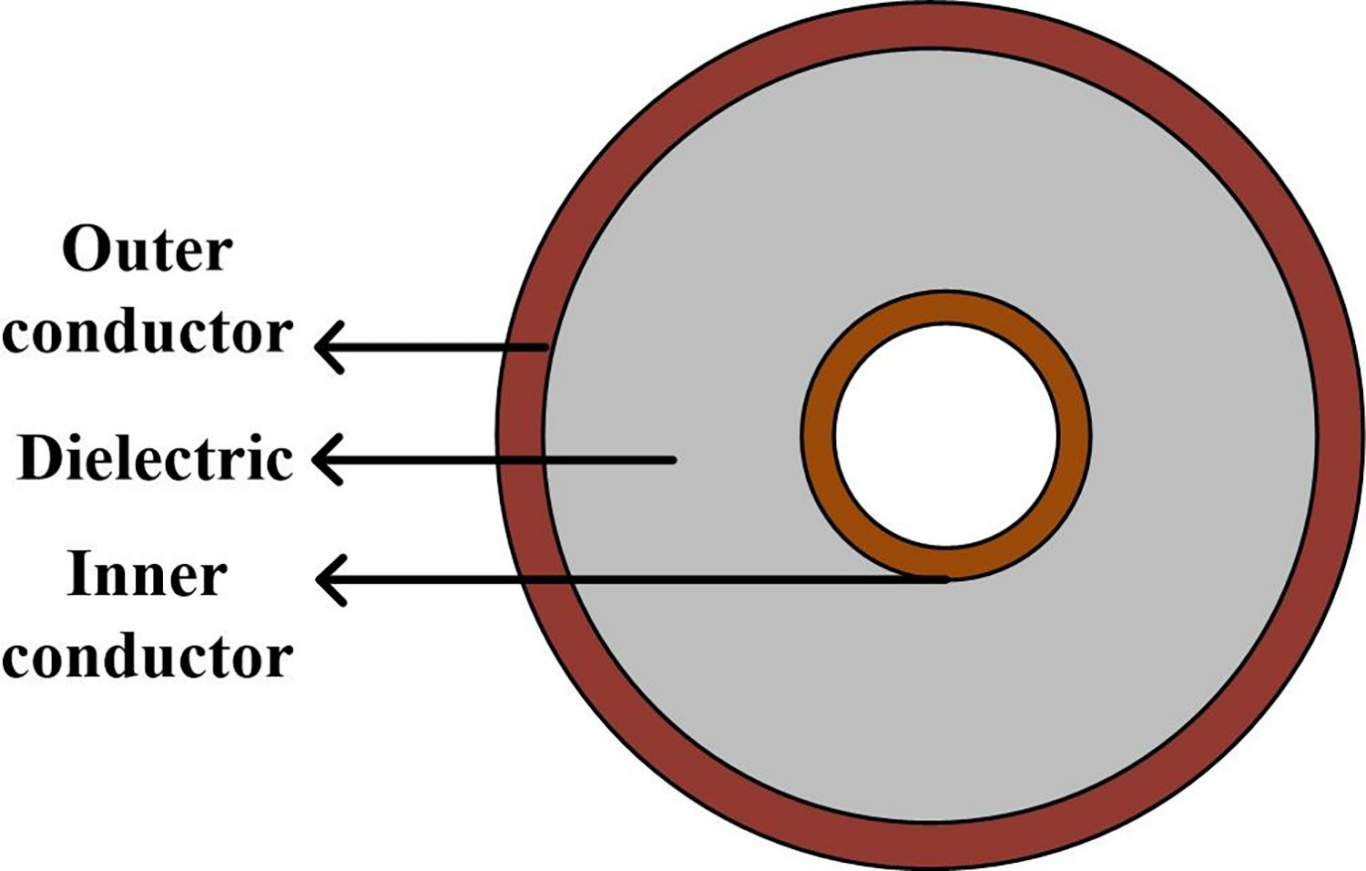
Cross-sectional structure of LCX.

**Fig 2:**
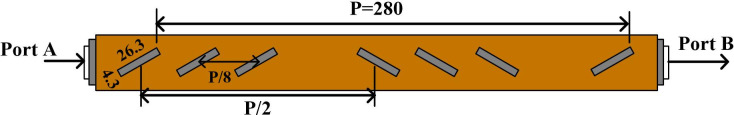
Geometric structure and parameters of LCX1.

**Fig 3:**
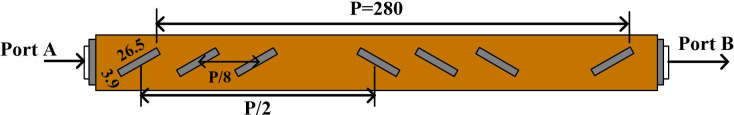
Geometric structure and parameters of LCX2.

The structure and geometries of the LCX1 and LCX2 are shown in Figs 1–3. We perform the LCXs simulation at 2100 MHz, 2600 MHz and 3400 MHz, which represent the main frequency point of 4G and 5G communication bands. The simulation results of the LCXs are shown in Table 1. It shows that, the transmission loss and voltage standing wave ratio (VSWR) of the LCX1 is lower than that of the LCX2, and the coupling loss is higher than that of the LCX2. It means no matter the emitted energy from the leaky gap or the received energy by receiver is reduced. But both their performances are better than the standard values, which could meet the requirements of the engineering.

**Table 1:** Performance simulation results of LCXs.

LCX	Frequency (MHz)	Transmission loss (dB/100m)		Coupling loss (dB)		VSWR	
		Standard value	Simulation value	Standard value	Simulation value	Standard value	Simulation value
**LCX2**	2100	10.75	4.15	74	64	1.3	1.088
	2600	13.9	6.15	68	66	1.3	1.225
**LCX1**	2100	6.0	2.28	72	72.9	1.3	1.009
	2600	8.0	3.38	76	75	1.3	1.049
	3400	11	9.45	80	66.2	1.3	1.080

### 2.2 Subway carriage model

**Fig 4:**
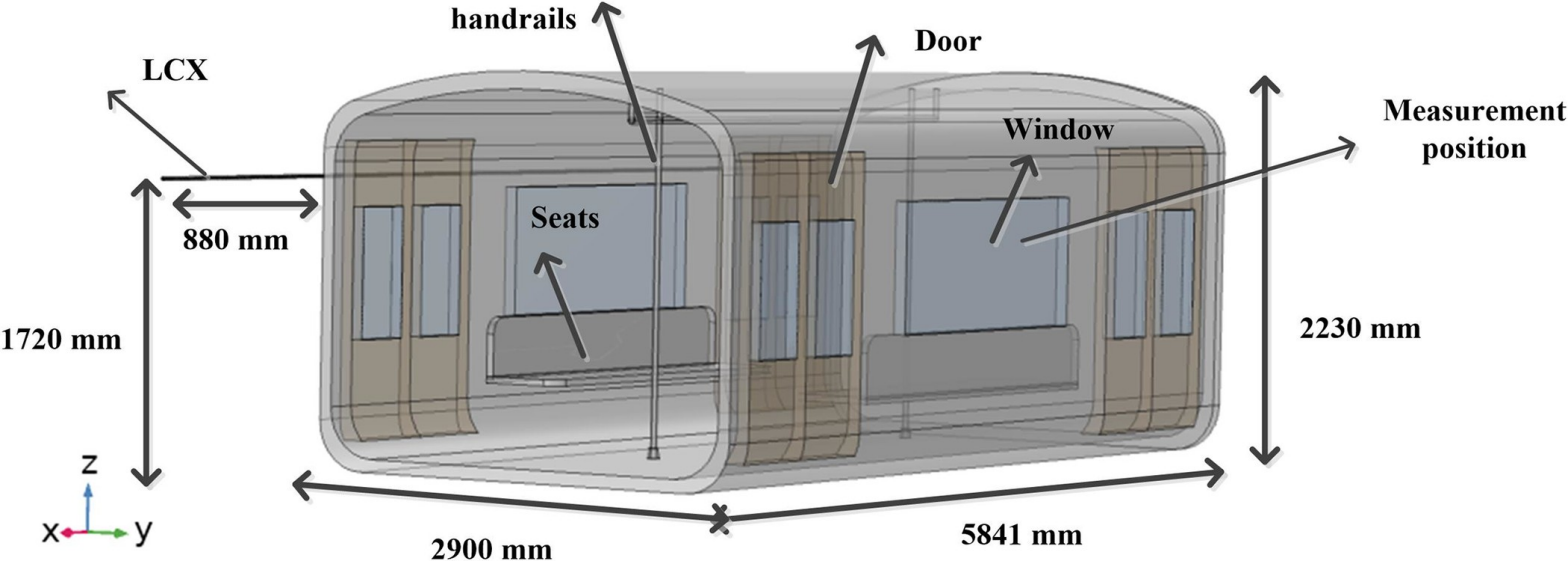
Subway carriage model. Carriage model and relative position with LCX.

The subway carriage in the tunnel model is established. Subway carriage and doors are made of aluminum alloy with the conductivity of 3.30×10^7^ S/m. LCX is placed above the side of the carriage, with a horizontal distance of 880 mm and a distance of 1720 mm from the bottom of the carriage, as shown in Fig 4.

### 2.3 Human models of the passengers

**Fig 5:**
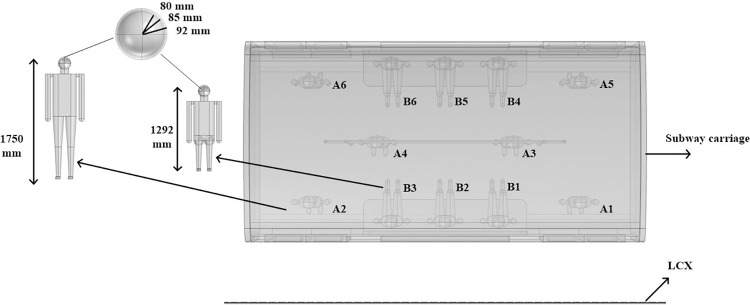
Human models of the passengers in the subway carriage. 6 standing passengers (marked as A1~A6) and 6 sitting passengers (marked as B1~B6).

There are 12 human models of passengers in the carriage, including 6 standing passengers (tagged with A_1_~A_6_) and 6 sitting passengers (tagged with B_1_~B_6_). The head of the human model is constructed with a three-layer spherical head model, which represents the brain, skull and scale, the geometry is shown in Fig 5.

The dielectric properties, mass density, and thermal parameters of the human tissues in the 2100 MHz, 2600 MHz and 3400 MHz frequencies are shown in Tables 2 and 3. The electrical conductivity and relative permittivity of the brain are taken as the average value of cerebrospinal fluid, white matter and grey matter, the trunk is taken as the average value of muscle, blood and bone. The dielectric properties of passenger body tissues are obtained through the 4-Cole-Cole extrapolation method. The mass density and thermal parameters of human tissues are obtained from reference [17].

**Table 2:** Dielectric parameters of human tissues.

Freq(Hz)	Conductivity(S/m)			Relative permittivity		
	2100	2600	3400	2100	2600	3400
**Scalp**	1.3075	1.5357	1.9655	38.431	37.845	37.092
**Skull**	0.50664	0.64129	0.876145	15.2775	14.836	14.1885
**Brain**	1.92483	2.2685	2.9169	50.958	50.225	49.10133
**Trunk**	1.197045	1.450245	1.9206225	35.64225	35.05825	34.15825

**Table 3:** Mass density and thermal parameters of human tissues.

Tissue	ρ (kg/m^3^)	K (W/(m·°C))	C (J/(kg·°C))	ω_b_ (1/s)	Q_met_ (W/m^3^)
**Scalp**	1125	0.42	3600	0.02	1620
**Skull**	1990	0.37	3100	4.63×10^−4^	610
**Brain**	1038	0.53	3650	8.83×10^−3^	7100

### 2.4 EMF of subway carriage modeling and verification

To verify the reliability of the constructed numerical calculation model, we measured the electric field environment inside the carriage. The input power of LCX would fluctuate due to changes in wireless communication network utilization. In order to ensure the stability of testing, we used Probe software to measure Reference Signal Receiving Power (RSRP). During the metro operation, measurement data was obtained at 1-second intervals, resulting in a total of 2744 data points. The input impedance of the measurement system is 50 Ω, ignoring losses. The formula for calculating the electric field strength is as follows:

E=K+A+107+L (3)

where *K* is the antenna coefficient (dB), which is the ratio of electric field intensity to receiver port voltage, and *A* is the signal strength of the spectrum analyzer or electromagnetic analyzer (dBm). The difference between the measured voltage of 50 Ω measuring equipment and the digital amplitude reading is 107. If there is a cable connection between the receiving antenna and electromagnetic analyzer, *L* is the loss value of the connecting cable (dB). The antenna gain *G* is usually used to calculate the antenna coefficient *K*

$$K=20lg\left(\frac{9.76}{\lambda \sqrt{G}}\right)$$ (4)

where λ is the wavelength (m).

**Fig 6:**
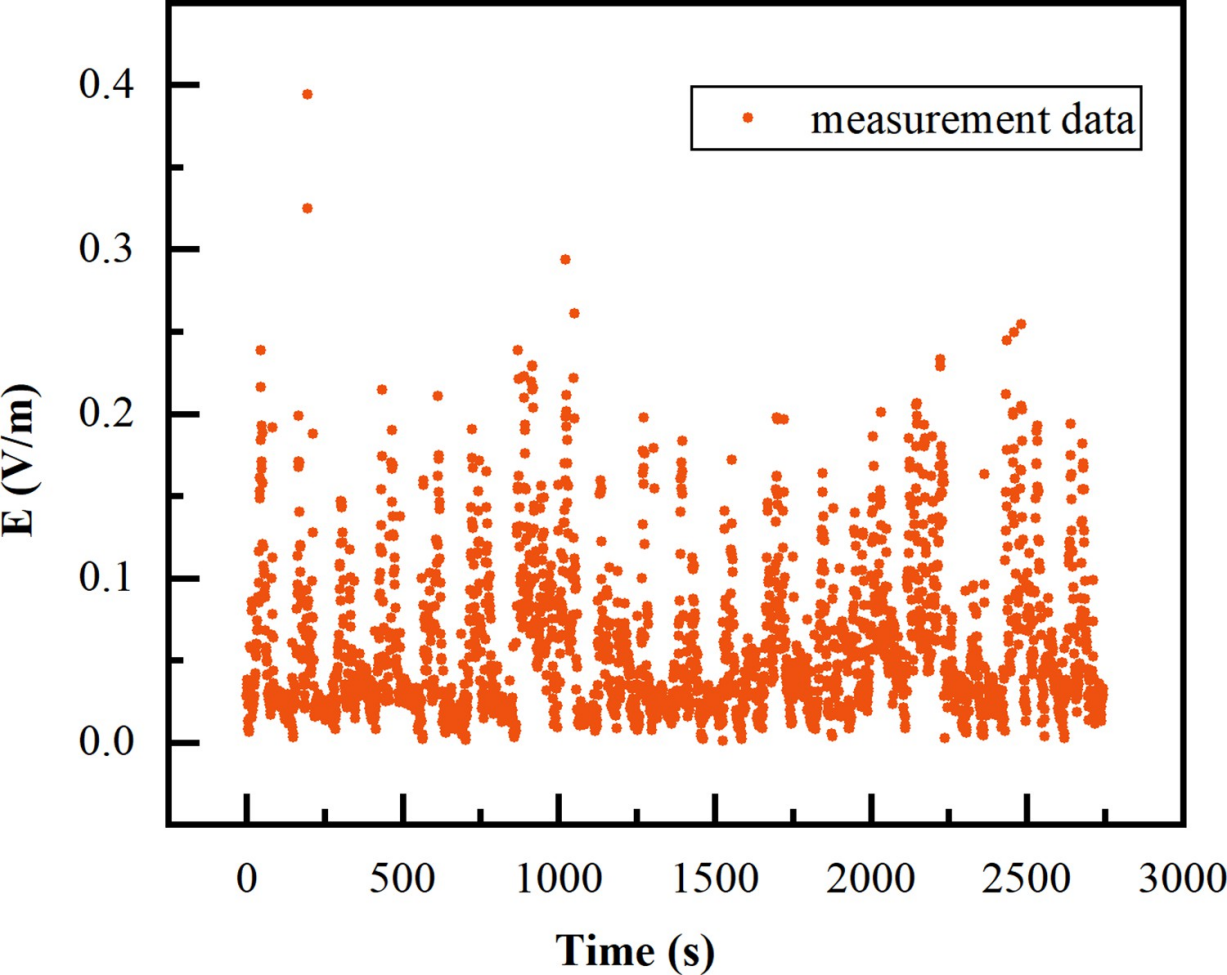
Measurement results. Scatter plot distribution of electric field measurement results.

**Fig 7:**
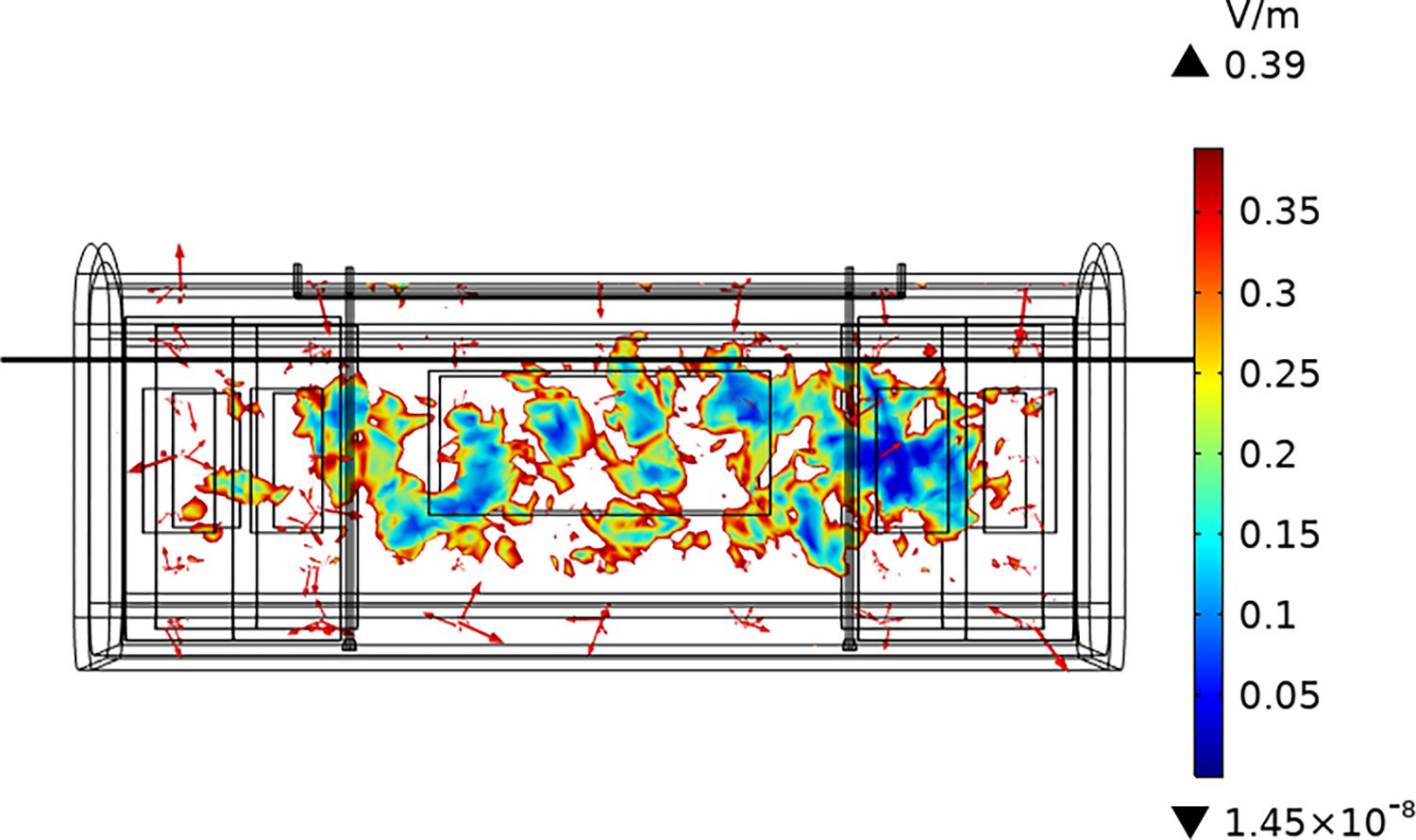
Distribution of simulated electric field values on the measurement point plane.

For verified the reliability of the electromagnetic environment in our constructed subway carriage, we converted the measured data points of signal power into electric field strength values through formula, and represent 2744 electric field intensity values in the form of a scatter plot, as shown in Fig 6. The electric field intensity simulated values in our proposed RF environment model focused on the range of 6.99×10^−4^ ~ 0.39 V/m as shown in Fig 7, which were basically consistent with the measured values. Therefore, the model of RF-EMF environment we have established can be used to study the electromagnetic exposure of passengers.

## 3. Safety evaluation of the passengers in the subway carriage

The radiation dose absorbed by 12 passengers are simulated by the COMSOL Multiphysics software, when different LCXs operated at different frequencies (case 1~case 5).

### 3.1 Analysis of the EMF dose

The maximum values of the SAR and induced electric fields (|***E***|) in the human model when exposed to the five different cases are listed in the Table 4. It shows that, the maximum SAR values are 3.81×10^−5^ W/kg and 1.04×10^−4^ W/kg when LCX2 worked at 2100 MHz and 2600 MH respectively. The maximum SAR values are 3.46×10^−5^ W/kg and 8.06×10^−5^ W/kg when LCX1 worked at 2100 MHz and 2600 MHz respectively.

**Table 4:** Physical quantity of 12 passengers.

LCX	Frequency (MHz)	Physical quantity	Position											
			**A**_**1**_	**A**_**2**_	**A**_**3**_	**A**_**4**_	**A**_**5**_	**A**_**6**_	**B**_**1**_	**B**_**2**_	**B**_**3**_	**B**_**4**_	**B**_**5**_	**B**_**6**_
**LCX2**	**2100MHz(case 1)**	***|E|*(V/m)**	0.2107	0.2020	0.1140	0.1107	0.0972	0.0719	0.2461	0.2926	0.2748	0.0995	0.1030	0.1092
		**SAR (W/Kg)**	1.92×10^−5^	1.78×10^−5^	5.70×10^−6^	5.38×10^−6^	4.11×10^−6^	2.24×10^−6^	2.62×10^−5^	3.81×10^−5^	3.28×10^−5^	4.39×10^−6^	4.62×10^−6^	5.46×10^−6^
	**2600MHz (case 2)**	***|E|*(V/m)**	0.2397	0.2655	0.1633	0.1678	0.1092	0.1293	0.2930	0.4459	0.3311	0.1637	0.2301	0.1094
		**SAR (W/Kg)**	2.85×10^−5^	3.84×10^−5^	1.61×10^−5^	1.96×10^−5^	5.75×10^−6^	7.30×10^−6^	4.47×10^−5^	1.04×10^−4^	6.55×10^−5^	1.85×10^−5^	5.82×10^−5^	9.32×10^−6^
**LCX1**	**2100MHz (case 3)**	***|E|*(V/m)**	0.1424	0.1638	0.1016	0.1012	0.0846	0.0651	0.2290	0.2661	0.2125	0.0854	0.0922	0.1077
		**SAR (W/Kg)**	9.53×10^−6^	1.19×10^−5^	4.51×10^−6^	4.11×10^−6^	3.15×10^−6^	2.05×10^−6^	2.28×10^−5^	3.46×10^−5^	1.98×10^−5^	3.33×10^−6^	3.54×10^−6^	5.16×10^−6^
	**2600MHz (case 4)**	***|E|*(V/m)**	0.2100	0.2200	0.1565	0.0970	0.1098	0.0948	0.3149	0.3924	0.2720	0.1132	0.1277	0.1073
		**SAR (W/Kg)**	2.45×10^−5^	2.52×10^−5^	1.29×10^−5^	5.36×10^−6^	6.46×10^−6^	4.62×10^−6^	5.27×10^−5^	8.06×10^−5^	3.91×10^−5^	6.72×10^−6^	8.60×10^−6^	6.06×10^−6^
	**3400MHz (case 5)**	***|E|*(V/m)**	0.2134	0.1545	0.1201	0.0730	0.0737	0.0648	0.3840	0.4830	0.5246	0.1119	0.1149	0.1171
		**SAR (W/Kg)**	1.40×10^−4^	7.47×10^−5^	2.69×10^−5^	1.49×10^−5^	1.73×10^−5^	1.23×10^−5^	1.18×10^−4^	1.67×10^−4^	1.91×10^−4^	8.74×10^−6^	9.19×10^−5^	1.10×10^−5^
**ICNIRP public restrictions**		***|E|*(V/m)**	2~300GHz: 61											
		**SAR (W/Kg)**	10MHz~10GHz: 0.08 (whole body), 2 (head)											

Besides that, all the passengers´exposure dose are reduced when the proposed LCX1 worked. In other words, the proposed LCX1 could reduce the radiation dose in the passengers. Compared the EMF exposure degree under the different frequencies of LCXs, the radiation dose would increase by the frequency rise, and the SAR value achieves 1.91×10^−4^ W/Kg when the frequency rise to 3400 MHz. The maximum values focus on passenger B_2_ and B_3_, who is sitting behind the window and near to the radiation sources.

### 3.2 Analysis of the SAR distribution

To understand the SAR distribution inside each tissue of the passenger, the distribution analysis is required. Figs 8–17 shows the distribution of SAR for passengers with the highest SAR value exposed to the five different cases respectively.

**Fig 8:**
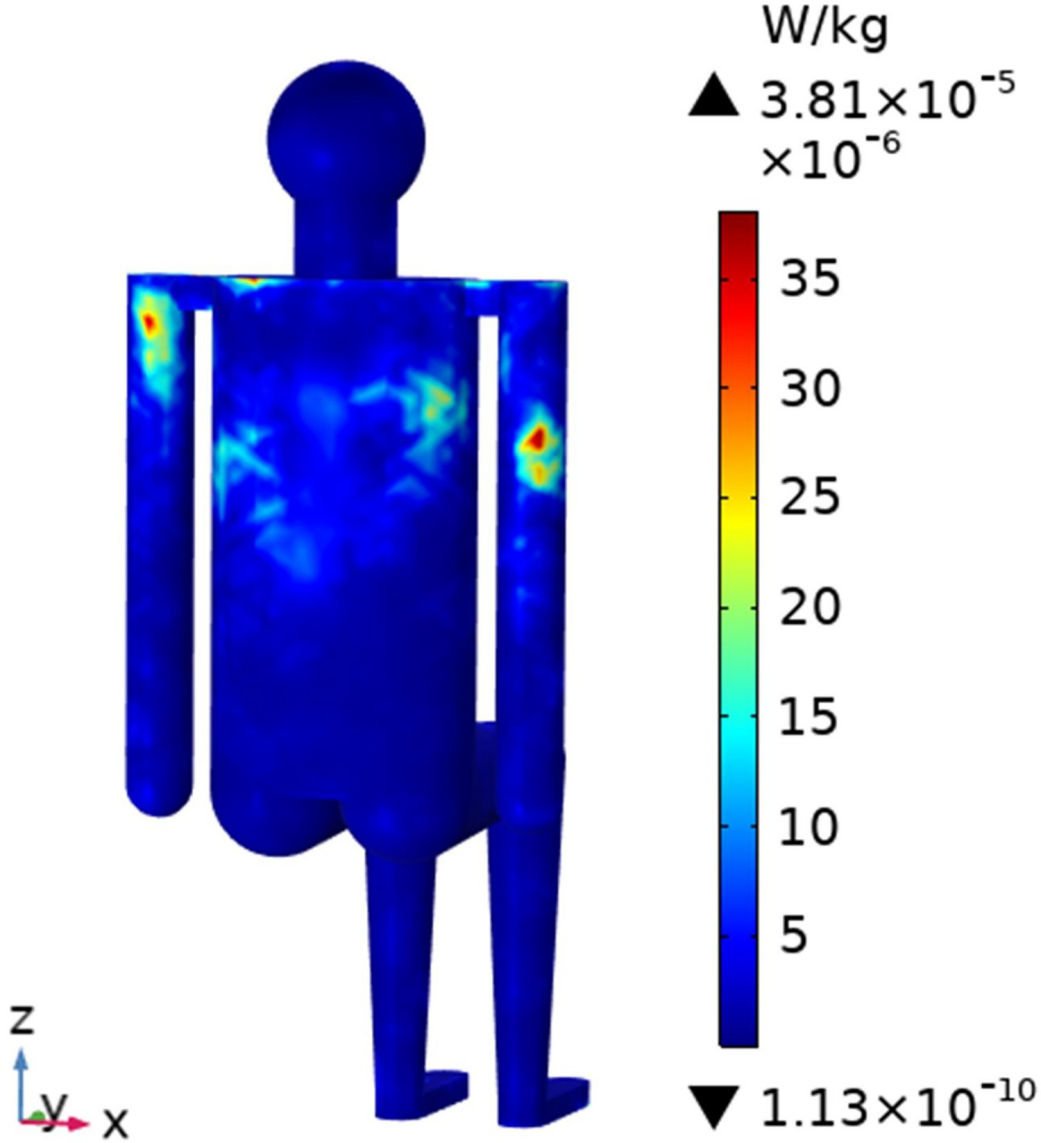
SAR distribution in passenger B2 when LCX2 works at 2100MHz.

**Fig 9:**
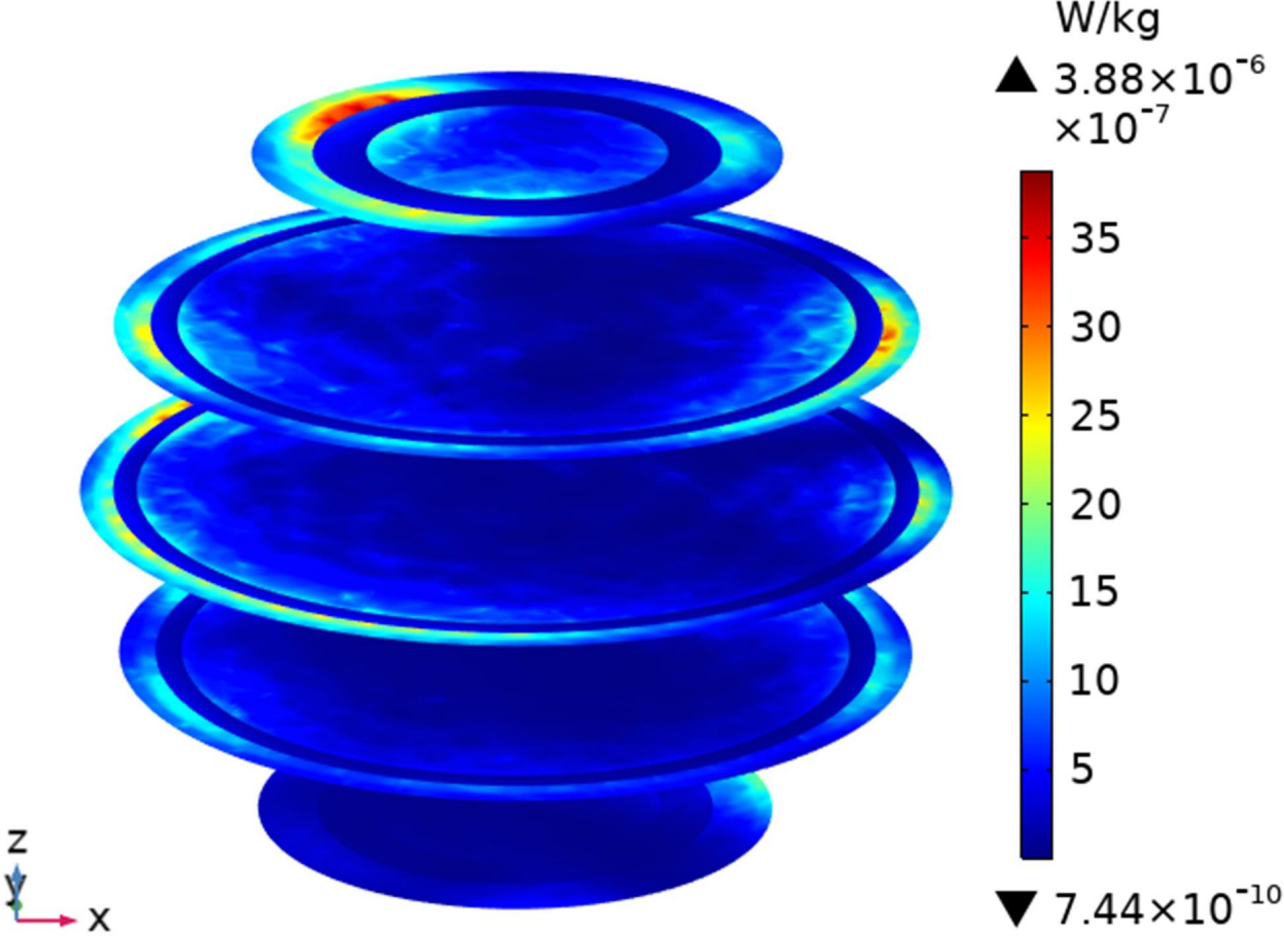
SAR distribution in the head section of passenger B2 when LCX2 works at 2100MHz.

**Fig 10:**
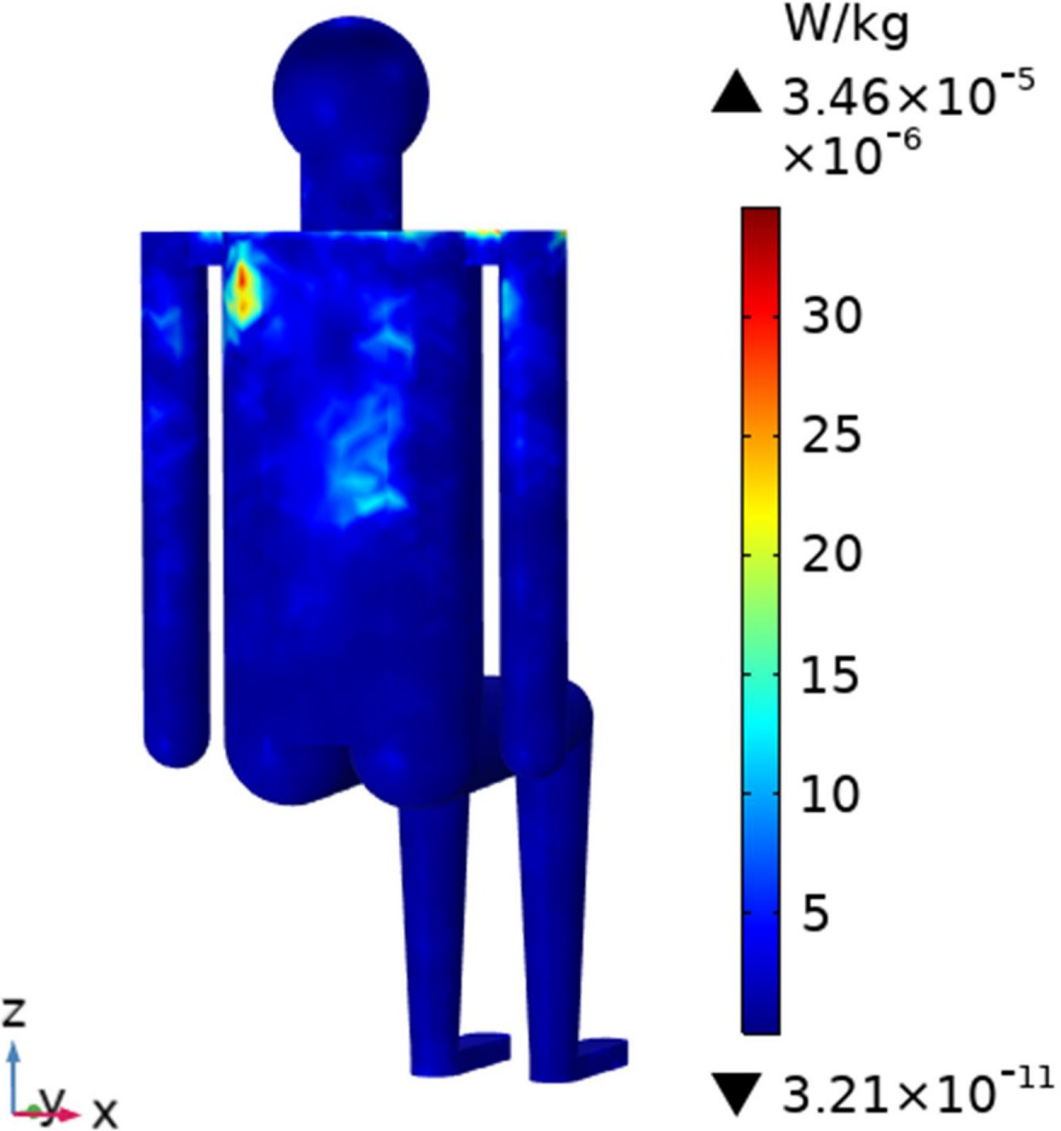
SAR distribution of passenger B2 when LCX1 works at 2100MHz.

**Fig 11:**
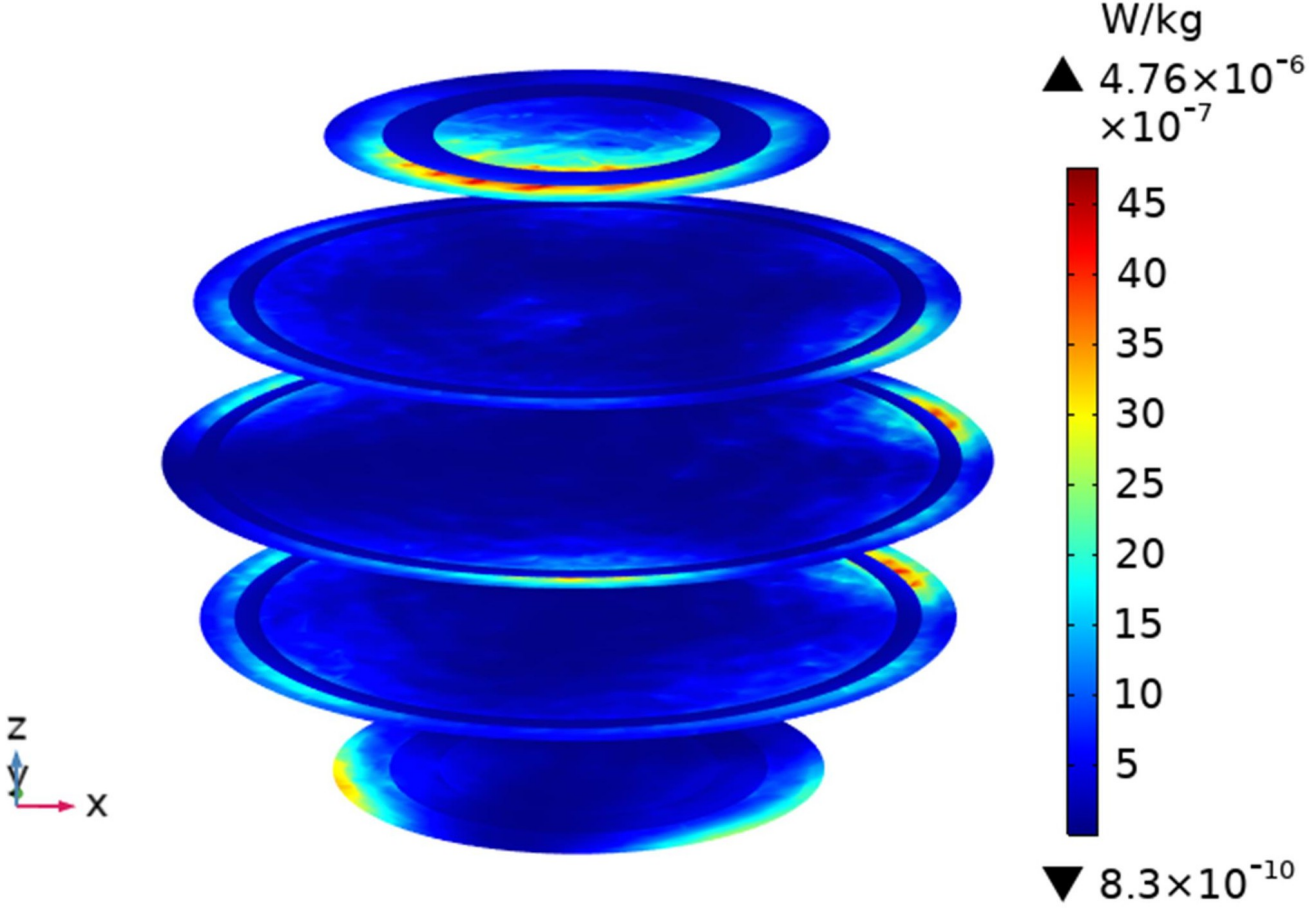
SAR distribution in the head section of passenger B2 when LCX1 works at 2100MHz.

**Fig 12:**
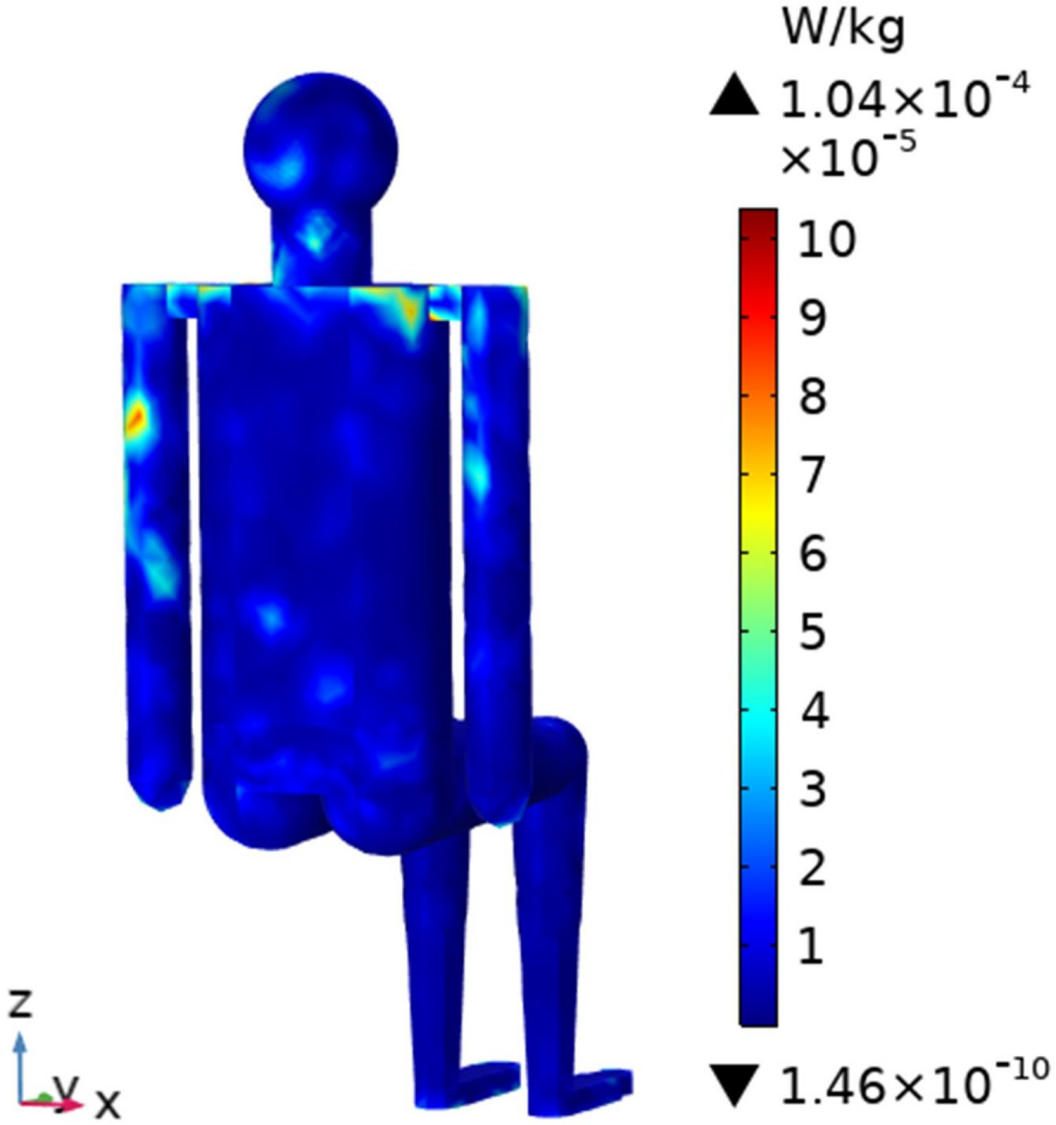
SAR distribution in passenger B2 when LCX2 works at 2600MHz.

**Fig 13:**
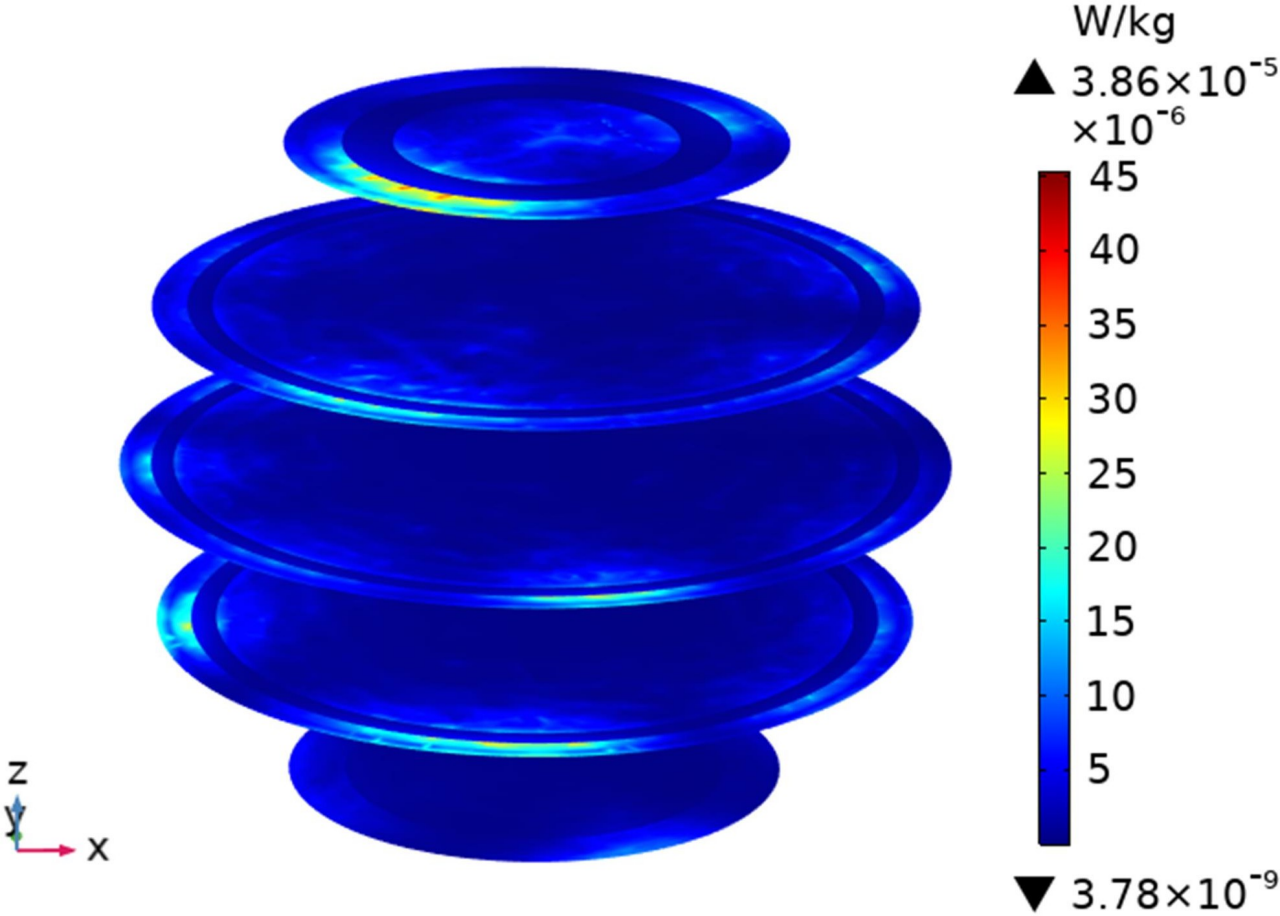
SAR distribution in the head section of passenger B2 when LCX2 works at 2600MHz.

**Fig 14:**
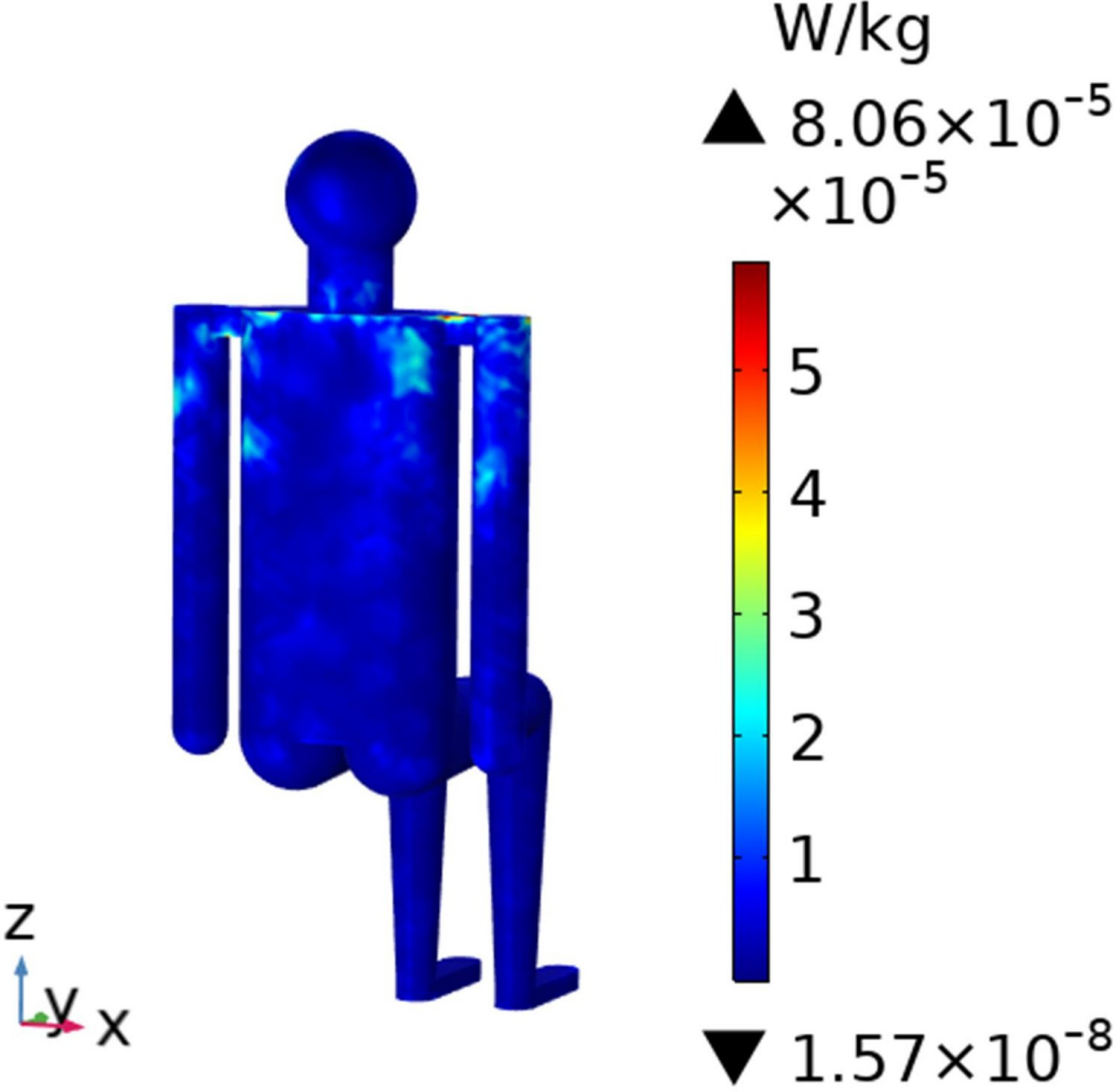
SAR distribution in passenger B2 when LCX1 works at 2600MHz.

**Fig 15:**
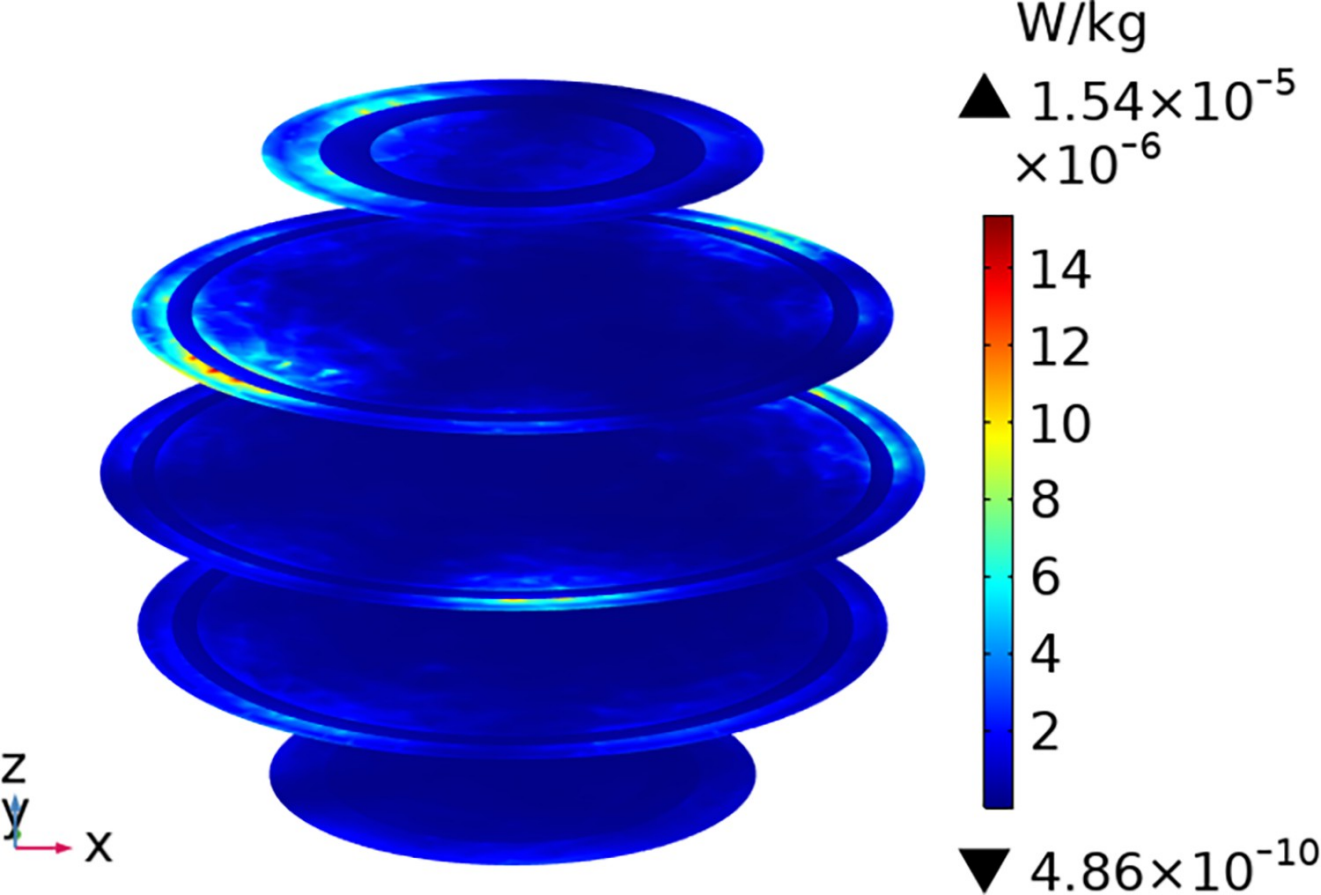
SAR distribution in the head section of passenger B2 when LCX1 works at 2600MHz.

**Fig 16:**
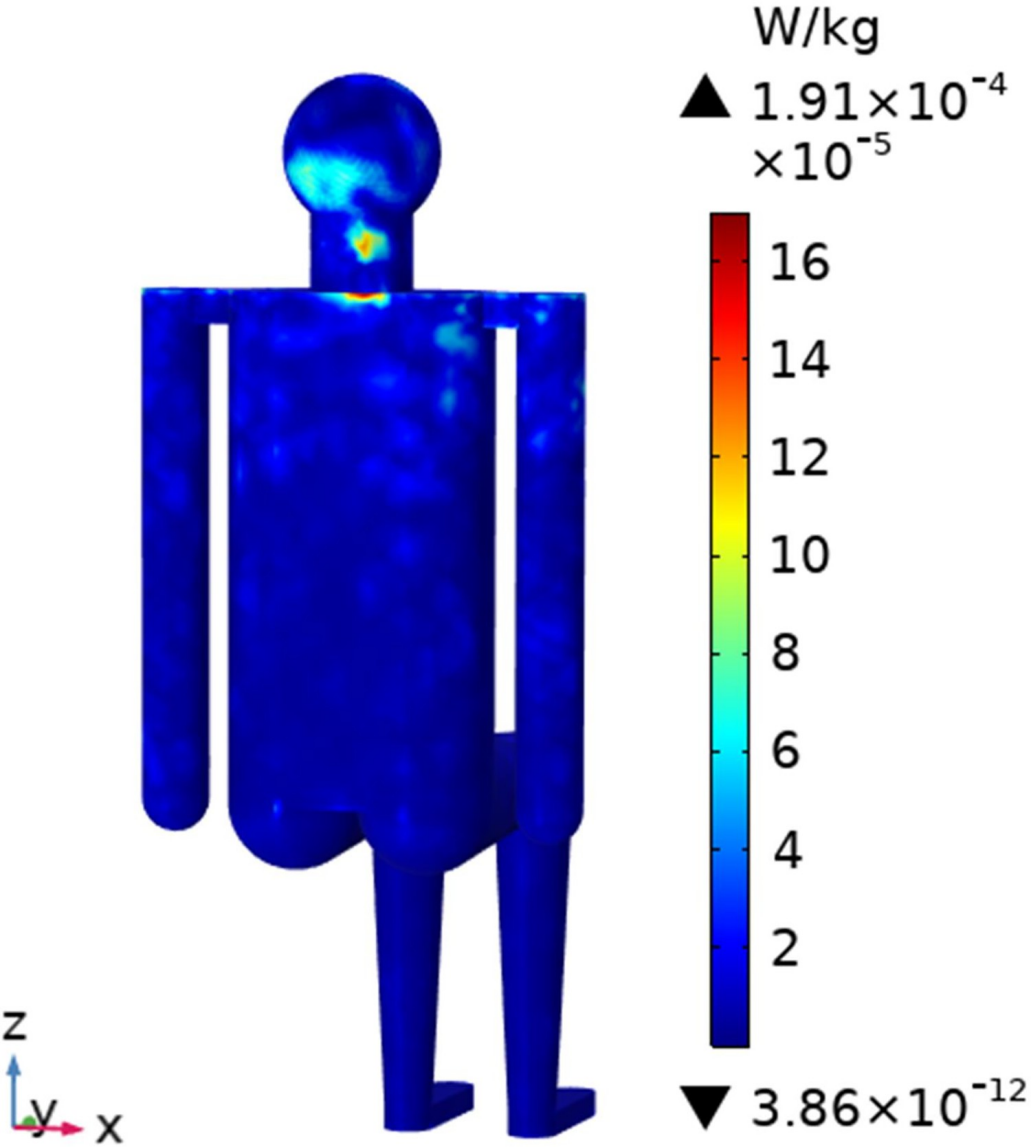
SAR distribution in passenger B3 when LCX1 works at 3400MHz.

**Fig 17:**
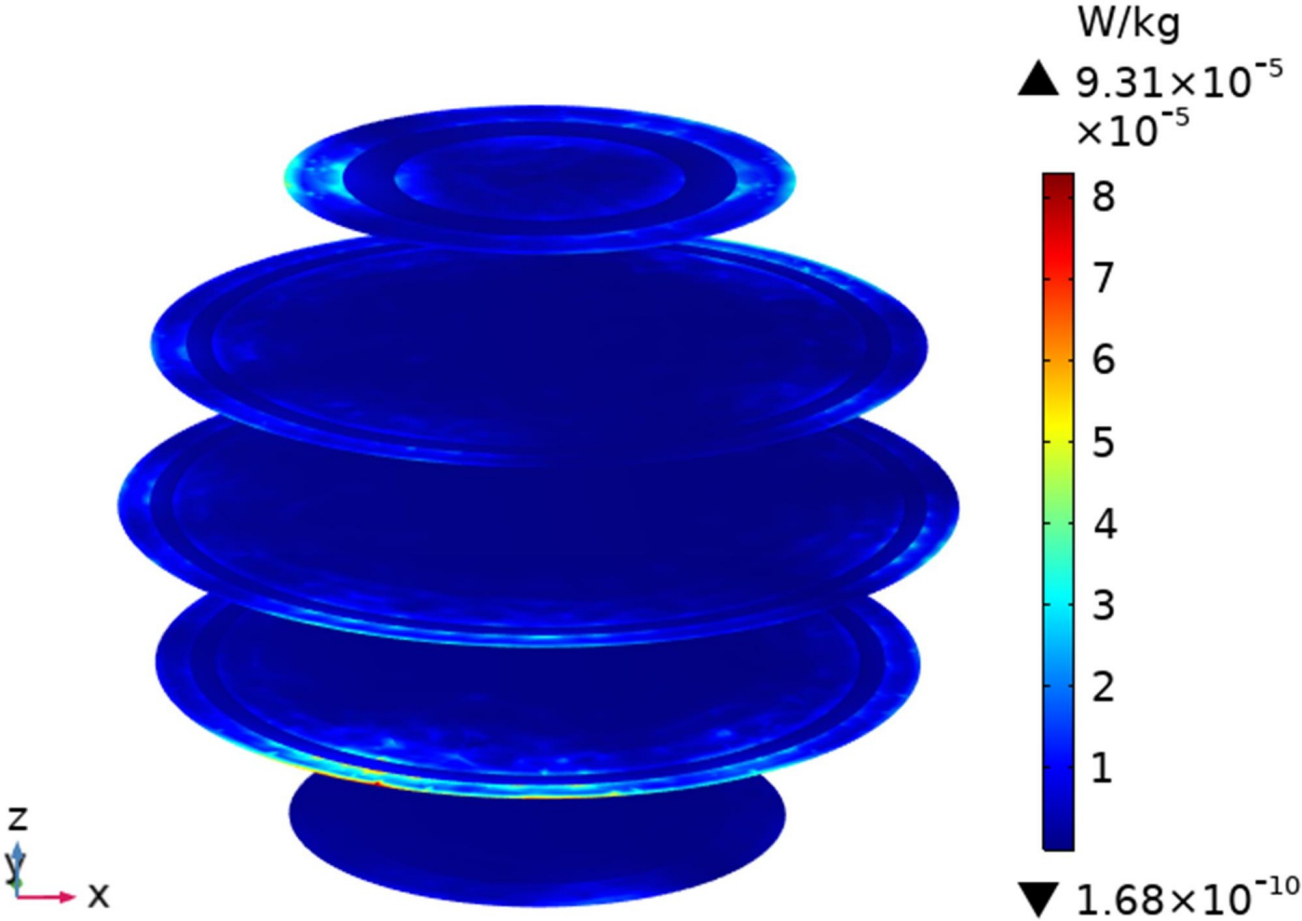
SAR distribution in the head section of passenger B3 when LCX1 works at 3400MHz.

When LCXs at the operation frequency of 2100 MHz, the SAR distribution by different LCXs is compared. When LCX2 is used as the exposure source, the electromagnetic energy absorbed by the passenger B_2_ is mainly distributed in the upper half of the arms. When LCX1 is used as the exposure source, the absorption is primarily concentrated in the back of passenger B_2_, and the maximum SAR of passengers is reduced by 9.19% compared to LCX2, as shown in Figs 8–11.

When LCXs at the operation frequency of 2100 MHz, the SAR distribution by different LCXs is compared. When LCX2 is used as the exposure source, the EMF dose absorbed by the passenger B_2_ is mainly distributed in the shoulder area. When LCX1 is used as the exposure source, the SAR is primarily distributed in the upper part of the arms and the back of the passenger B_2_, and the maximum SAR of passengers is reduced by 22.50% compared to LCX2, as shown in Figs 12–15.

When the LCX1 operates at 3400 MHz, the absorbed electromagnetic dose by the passenger B_3_ shows a scatter distribution, mainly occurring in the shoulder and neck areas as shown in Figs 16 and 17.

Comparing the SAR distribution of brain sections at different frequencies, it can be concluded that as the frequency increases, the penetration of electromagnetic waves decreases. The EMF energy that reaches the brain layer is reduced and is mostly absorbed by the scalp layer, due to the electromagnetic waves would attenuation faster as the frequency increased.

### 3.3 Analysis of the temperature rise of the LCX1

**Fig 18:**
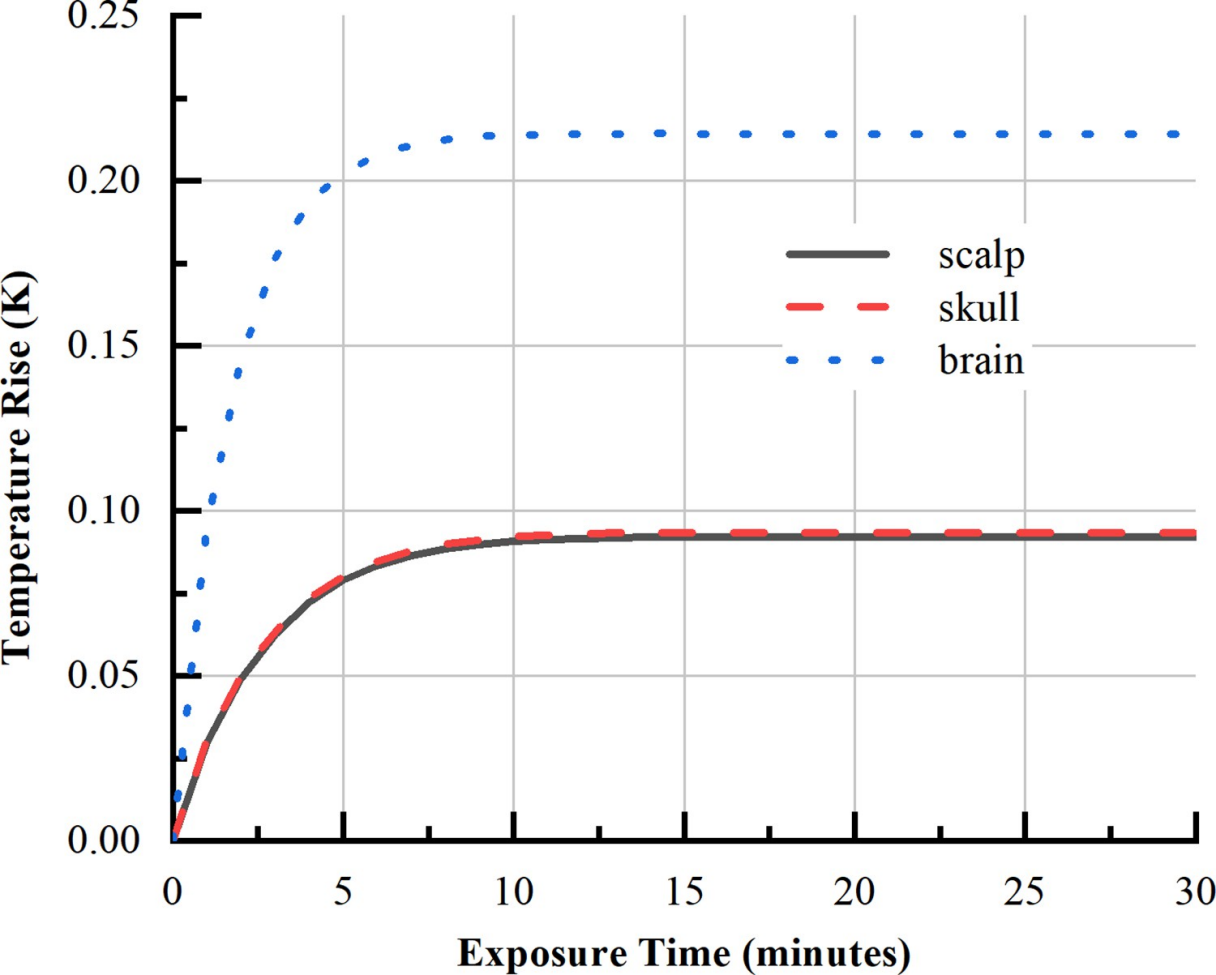
Temperature rise vs exposure time when LCX1 operating at 3400MHz. Changes in temperature rise of the three tissues in the head within 30 minutes.

In order to evaluate the safety of the thermal effects caused by LCX, we simulated the temperature rise during long time exposure for the exposure scenario which caused the highest SAR value (i.e. LCX1 operating at 3400 MHz). It can be observed that the peak temperature increases exponentially over the first 5–6 minutes, and then the rate slows down as shown in Fig 18. The maximum temperature rise of the scalp, skull, and brain tissues is 0.092 k, 0.093 k and 0.214 k respectively.

### 3.4 Analysis of the input power of the LCX1

**Fig 19:**
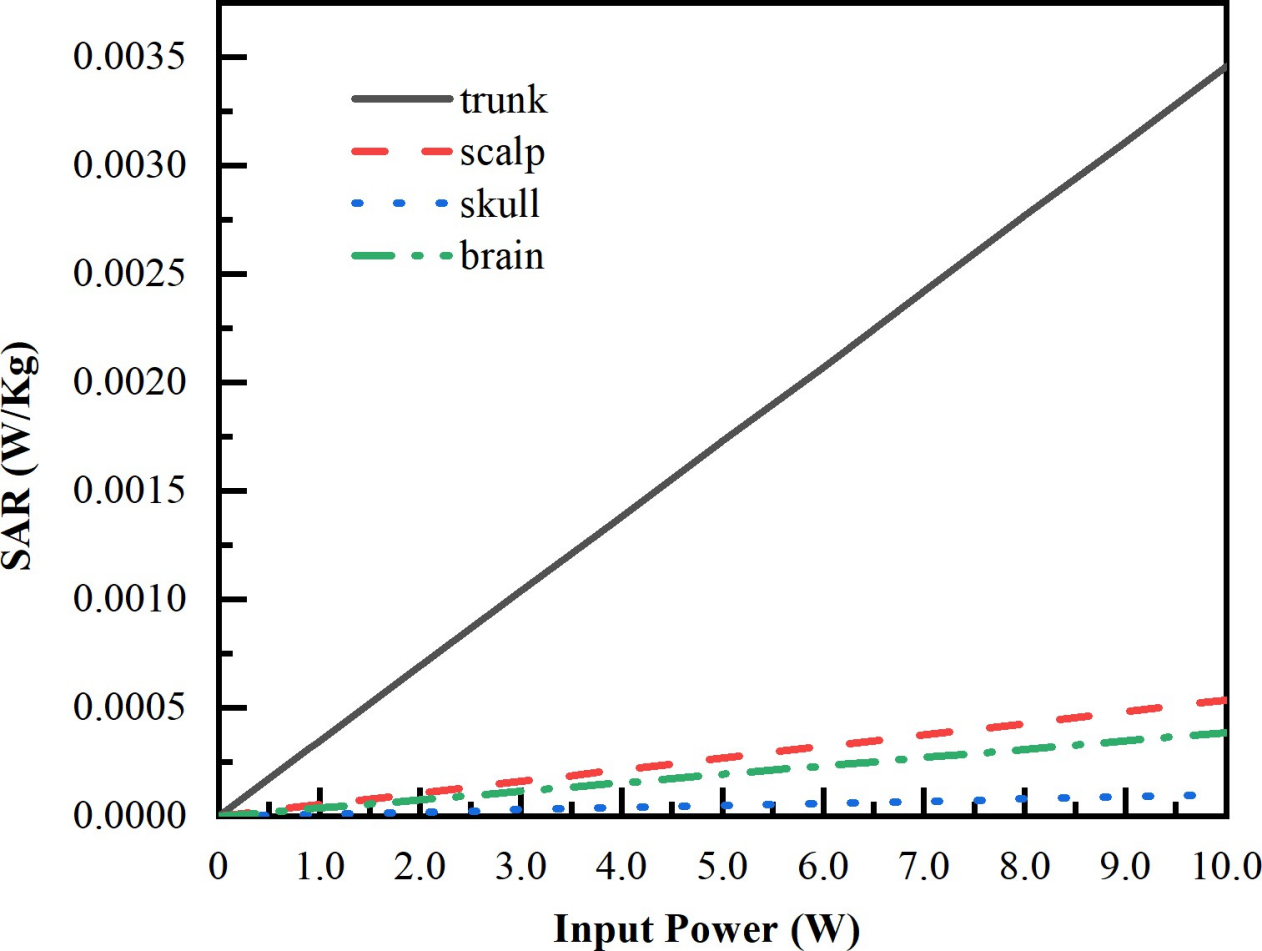
SAR value vs input power under LCX1 at 2100 MHz. Tendency of SAR in different input powers of LCX1 at 2100 MHz.

**Fig 20:**
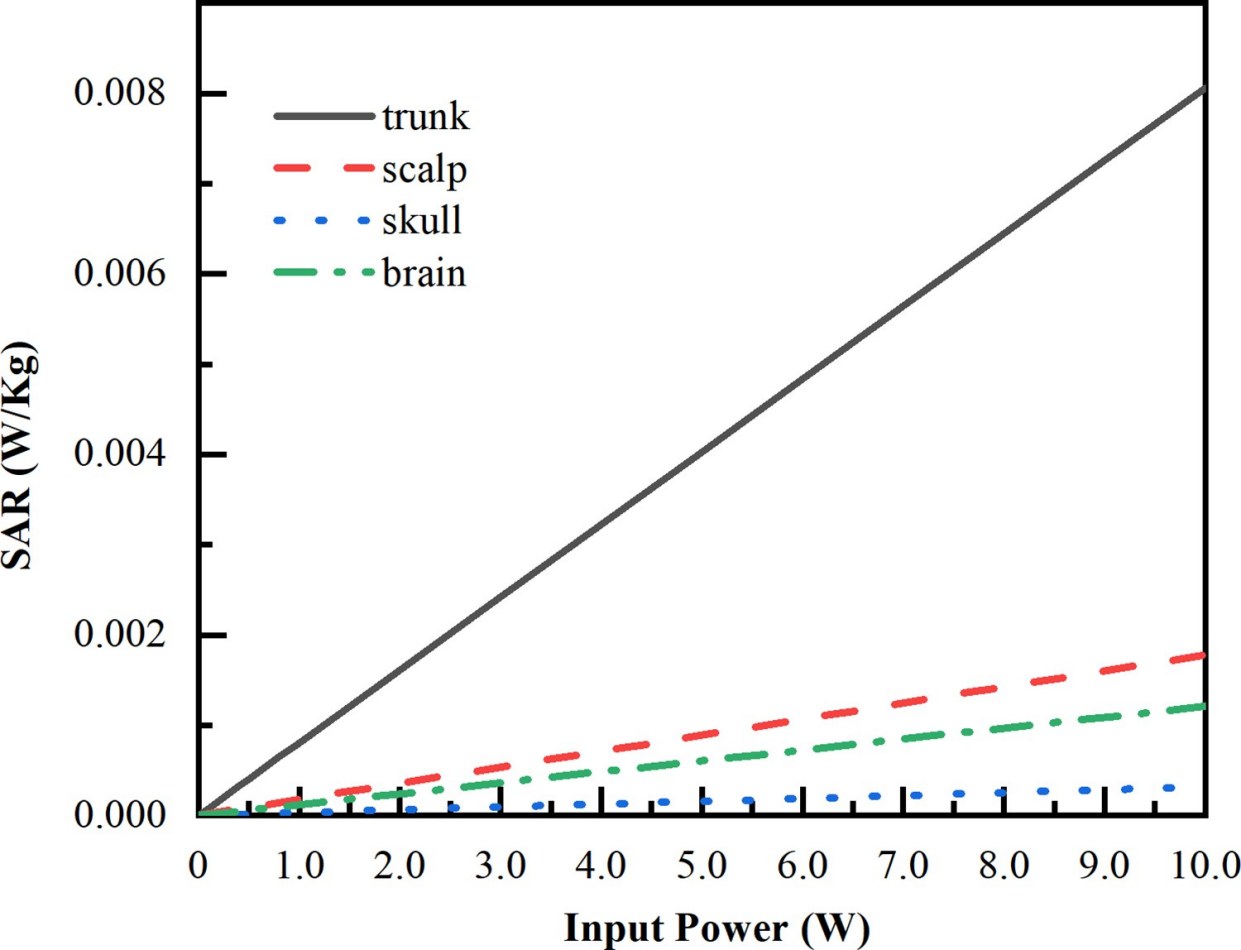
SAR value vs input power under LCX1 at 2600 MHz. Tendency of SAR in different input powers of LCX1 at 2600 MHz.

**Fig 21:**
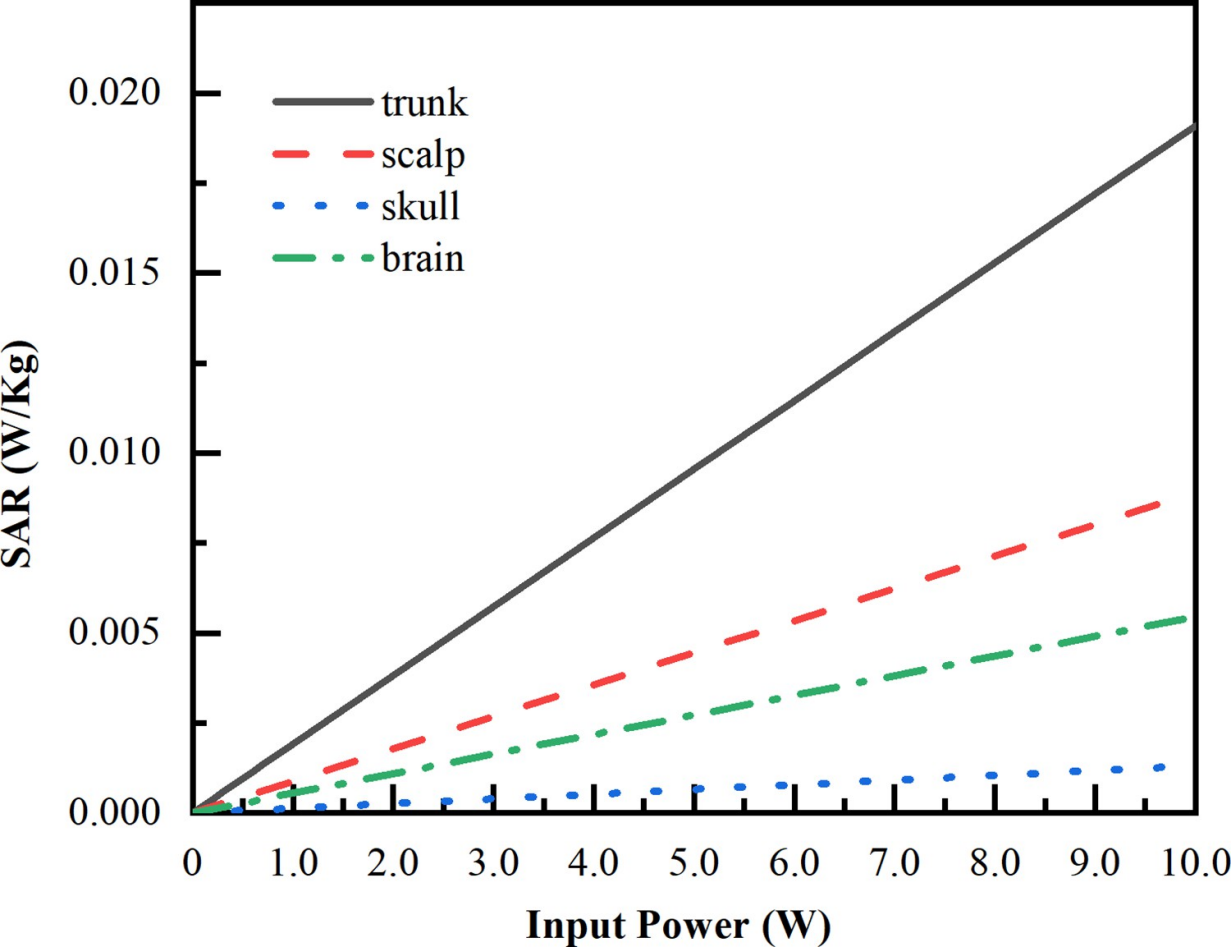
SAR value vs input power under LCX1 at 3400 MHz. Tendency of SAR in different input powers of LCX1 at 3400 MHz.

Since the input power of LCX would be adjusted by the subway real-time communication requirement, we simulate the SAR variation in different tissues of the human model as the input power varies from 0.1 W to 10 W at each frequency (2100 MHz, 2600 MHz, 3400 MHz). The simulated variation results are shown in Figs 19–21. The maximum SAR variation appears in trunk with the maximum SAR of 1.91×10^−2^ W/kg, followed by the scalp with the maximum SAR of 8.91×10^−3^ W/kg, brain with the maximum SAR of 5.45×10^−3^ W/kg, and skull with the maximum SAR of 1.31×10^−3^ W/kg. It should be noted that, the maximum SAR value of the passengers linearly increases in response as the input power of LCX1 increases. Besides that, as the corresponding frequency increases, the more significant differences of SAR absorption in the different tissues.

## 4.Conclusion

This study analyze the distribution of induced electric field and SAR on different passengers in the subway carriage under the civil communication system. Particularly, we delve into the impact of radiation caused by different exposure sources (LCX1 and LCX2) with diverse radiated characteristics. The conclusion is as follows:

1. Compared with LCX2, the transmission loss of proposed LCX1 is decrease 45%, and coupling loss is increase 14%. When LCX1 operates at 2100 MHz and 2600 MHz, the electromagnetic dose absorbed by passengers is reduced by 9.19% and 22.50% compared to LCX2, respectively. This means that LCX1 radiates less energy outward while maintaining communication quality, which can lower the radiation impact on passengers.
2. The maximum SAR value occurs when LCX1 operates at 3400 MHz, with the value of 1.91 × 10^−4^ W/Kg. When the input power increases from 0.1 W to 10 W, the maximum SAR increases from 1. 91 × 10^−4^ W/kg increased to 1.91 × 10^−2^ W/kg, below the ICNIRP public restrictions. The maximum SAR value of passengers is directly proportional to the input power of LCX.
3. When LCX1 operates at 3400 MHz, the passenger’s temperature rise is the highest, with the value of 0.214 k. The peak temperature first increases exponentially and then slows down. In the first 0–5 minutes, the temperature rises to 0.200 k, and then changes slowly from 5–10 minutes. After about 10 minutes, the temperature rise reaches a stable state.

In summary, under the civil communication system, passengers are safe when compared to the ICNIRP public exposure limit. Furthermore, by modifying the radiation performance of the exposure source, the electromagnetic impact on passengers can be reduced, offering a novel approach to electromagnetic protection.
